# Haemodynamic significance of extrinsic outflow graft stenoses during HeartMate 3™ therapy

**DOI:** 10.1093/ehjimp/qyae082

**Published:** 2024-08-29

**Authors:** Linus Ohlsson, Mårten Sandstedt, Joanna-Maria Papageorgiou, Anders Svensson, Ann Bolger, Éva Tamás, Hans Granfeldt, Tino Ebbers, Jonas Lantz

**Affiliations:** Department of Cardiothoracic and Vascular Surgery, Linköping University, 581 83 Linköping, Sweden; Department of Health, Medicine and Caring Sciences, Linköping University, 581 83 Linköping, Sweden; Center of Medical Image Science and Visualization (CMIV), Linköping University, 581 83 Linköping, Sweden; Center of Medical Image Science and Visualization (CMIV), Linköping University, 581 83 Linköping, Sweden; Department of Radiology in Linköping, Linköping University, Linköping, Sweden; Department of Cardiology in Linköping, Linköping University, Linköping, Sweden; Department of Cardiothoracic and Vascular Surgery, Linköping University, 581 83 Linköping, Sweden; Department of Health, Medicine and Caring Sciences, Linköping University, 581 83 Linköping, Sweden; Department of Health, Medicine and Caring Sciences, Linköping University, 581 83 Linköping, Sweden; Department of Medicine, University of California San Francisco, San Francisco, CA, USA; Department of Cardiothoracic and Vascular Surgery, Linköping University, 581 83 Linköping, Sweden; Department of Health, Medicine and Caring Sciences, Linköping University, 581 83 Linköping, Sweden; Center of Medical Image Science and Visualization (CMIV), Linköping University, 581 83 Linköping, Sweden; Department of Cardiothoracic and Vascular Surgery, Linköping University, 581 83 Linköping, Sweden; Department of Health, Medicine and Caring Sciences, Linköping University, 581 83 Linköping, Sweden; Department of Health, Medicine and Caring Sciences, Linköping University, 581 83 Linköping, Sweden; Center of Medical Image Science and Visualization (CMIV), Linköping University, 581 83 Linköping, Sweden; Department of Health, Medicine and Caring Sciences, Linköping University, 581 83 Linköping, Sweden; Center of Medical Image Science and Visualization (CMIV), Linköping University, 581 83 Linköping, Sweden

**Keywords:** LVAD, HeartMate 3, photon-counting CT, CFD, haemodynamics

## Abstract

**Aims:**

The HeartMate 3 (HM3) implantable left ventricular assist device connects the left ventricle apex to the aorta via an outflow graft. Extrinsic obstruction of the graft (eOGO) is associated with serious morbidity and mortality and recently led to a Food and Drug Administration Class 1 device recall of HM3. This study aimed to provide a better understanding of the haemodynamic impact of extrinsic stenoses.

**Methods and results:**

Computed tomography (CT) images of two retrospectively identified patients with eOGO (29 and 36% decrease in cross-sectional area, respectively, by radiological evaluation) were acquired with a novel photon-counting CT system. Numerical evaluations of haemodynamics were conducted using a high-fidelity 3D computational fluid dynamics approach on both the patient-specific graft geometries and in two virtually augmented stenotic severities and three device flows. Visual analysis identified increased velocity, pressure, and turbulent flow in the outer anterior curvature of the outflow graft; however, changes in graft pressure gradients were slight (1–9 mmHg) across the range of stenosis severities and flow rates tested.

**Conclusion:**

Evidence of eOGO during HM3 support and the recent device recall can provoke clinical apprehension and interventions. The haemodynamic impact of a stenosis detected visually or by quantification of cross-sectional area reduction may be difficult to predict and easily overestimated. This numerical study suggests that, for clinically encountered flow rates and stenosis severities below 61% in cross-sectional area decrease, eOGO may have low haemodynamic impact. This suggests that patients without symptoms or signs consistent with haemodynamically significant obstruction might be managed expectantly.

## Graphical Abstract

**Graphical Abstract:**
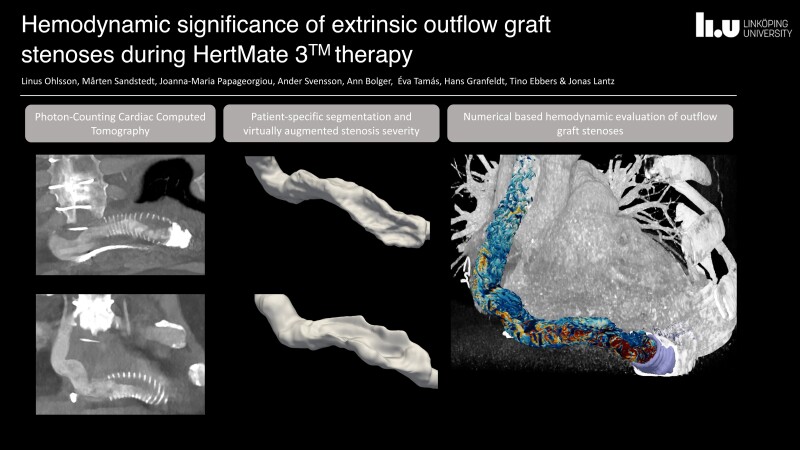

## Introduction

Heart failure is a global health concern, leading to millions of deaths annually.^1^ Mechanical circulatory support is an important tool in the face of cardiac donor scarcity.^2^ In the realm of long-term mechanical circulatory support, the HeartMate 3™ (HM3) (Abbott Laboratories, Lake Forest, IL), a fully magnetically levitated centrifugal flow pump, has seen increasing use in recent years.^3^ The significant benefits of the HM3 include enhanced haemocompatibility and fewer thromboembolic incidents compared with its predecessors.^4,5^ However, development of extrinsic obstruction of the outflow graft (eOGO) can occur during therapy. This complication was first shown in two case studies of patients admitted for congestive symptoms who had developed a proximal narrowing of the outflow graft at the location of the bend relief, which is an impermeable collar surrounding the porous graft near its attachment to the left ventricular (LV) apex.^6,7^ In these cases, the stenosis resulted in near-complete occlusion and consequent haemodynamic instability, necessitating urgent cardiac transplantation. Pathology-based examination of explanted material showed thrombus-like material between the graft and the surrounding bend relief.

A subsequent multicentre study^8^ involving 17 cardiac centres retrospectively analysed HM3 recipients (*n* = 2018) and defined extrinsic OGO as >25% decrease in cross-sectional area (CSA) based on different imaging methods [percutaneous angiography, computed tomography (CT), or intravascular ultrasound]. Sixty-two cases were identified with an increasing incidence at 1, 2, 3, 4, and 5 years of support of 0.6, 2.8, 4.0, 5.2, and 9.1%, respectively. While the majority presented with low flow alarms or dyspnoea, six patients were asymptomatic. Of 62 patients, 9 were observed, 27 underwent surgical revision, 15 underwent percutaneous stent implantation, 8 received heart transplantation, and 2 died before intervention. One patient underwent surgical revision and later stent implantation. The mortality with therapeutic intervention was 9/53 (17.0%). Due to this serious mortality, the U.S. Food and Drug Administration (FDA) recently published a Class 1 device recall of the HM3 on 22 March 2024.^9^

In general, stenoses of blood vessels and outflow regions generate a pressure gradient, resulting in decreased organ perfusion, increased load on the heart, and patient symptoms.^10,11^ When a significant portion of a left ventricular assist device (LVAD) patient’s cardiac output passes through the outflow graft, an eOGO would have similar effects. There are limited non-invasive approaches for assessing haemodynamic conditions in the outflow graft. Transthoracic ultrasound inadequately visualizes graft blood flow, and flow magnetic resonance imaging (MRI) is not feasible during HM3 support. However, our research group and others have previously demonstrated that numerical models can quantify various haemodynamic parameters in stenoses within the cardiovascular system with good agreement with *in vivo* measurements and provide additional information that cannot be obtained by other modalities.^12–14^

This study aimed to provide a better understanding of the haemodynamic impact of significant eOGO (as previously defined as >25% by Wert *et al.*) during HM3 support by using state-of-the-art photon-counting CT (PCCT). Advantages of PCCT over conventional CT include decreased electronic noise, improved spectral and spatial resolution, increased contrast-to-noise ratio, and reduction of metal artefacts.^15,16^ From these images, we aimed to numerically investigate haemodynamic conditions in patient-specific conditions and with virtually augmented stenotic severities and device flow.

Two baseline cases were identified within a clinical trial (NCT 06115096). The overall research project has been approved by the Swedish ethics review authority (reference number: 2022-06934-01). All research subjects, including the two cases within this study, have provided written consent.

## Methods

### Baseline cases

Cases 1 and 2 were males between 65 and 75 years of age who had an HM3 device implanted 4 and 7 years earlier, respectively. The patients were clinically stable without signs of congestion, haemodynamic impairment, or low flow alarms. The HM3 of Case 1 generated 4.9 L/min at 5700 rpm at the time of the CT and the HM3 of Case 2 generated 3.8 L/min at 5400 rpm.

### CT image acquisition and reconstructions

Cardiac CT scans utilizing the CE-marked PCCT system NAETOM Alpha (Siemens Healthineers, Erlangen, Germany) were retrospectively obtained from the two asymptomatic patients with radiologically identified outflow graft stenoses. The heart rates were 54 bpm (Case 1) and 58 bpm (Case 2) at the time of the scans. The acquisitions were performed at 120 kVp with image quality (IQ) levels set at 70 and spiral pitch factor of 0.17. Care Dose4D automatic exposure control was used. The matrix was 512 × 512. An iodine contrast medium, Omnipaque 350 mgI/mL (GE Healthcare, Chicago, IL, USA), was deployed, modulated with a saline dilution (Case 1: 110 mL; Case 2: 109.4 mL). Virtual mono-energetic reconstructions were exported at 110 keV. Kernels Bv40 (radiological evaluation) and Bv56 (segmentation) combined with a slice thickness of 0.4 mm (segmentation) and 1.5 mm (radiological evaluation) were all exported. Increments of 1.0 were employed over all reconstructions. Reconstructions in best diastolic phase were used in the radiological evaluation.

### Radiological evaluation

Specific reconstructions were qualitatively selected from the available options based on where a suspected extrinsic stenosis could best be delineated in relation to the metallic artefacts observed. Visual grading of stenotic significance according to preserved lumen diameter and CSA at 20 and 40 mm from the HM3 outflow tract was performed by two radiologists, specialized in cardiothoracic imaging with 20 and 40 years of radiological experience, respectively, using multiplanar reconstruction imaging in the clinically available software, Sectra Workstation IDS7 24.1 (Linköping, Sweden).

### Patient-specific geometry and surface deformation

**Figure 1:**
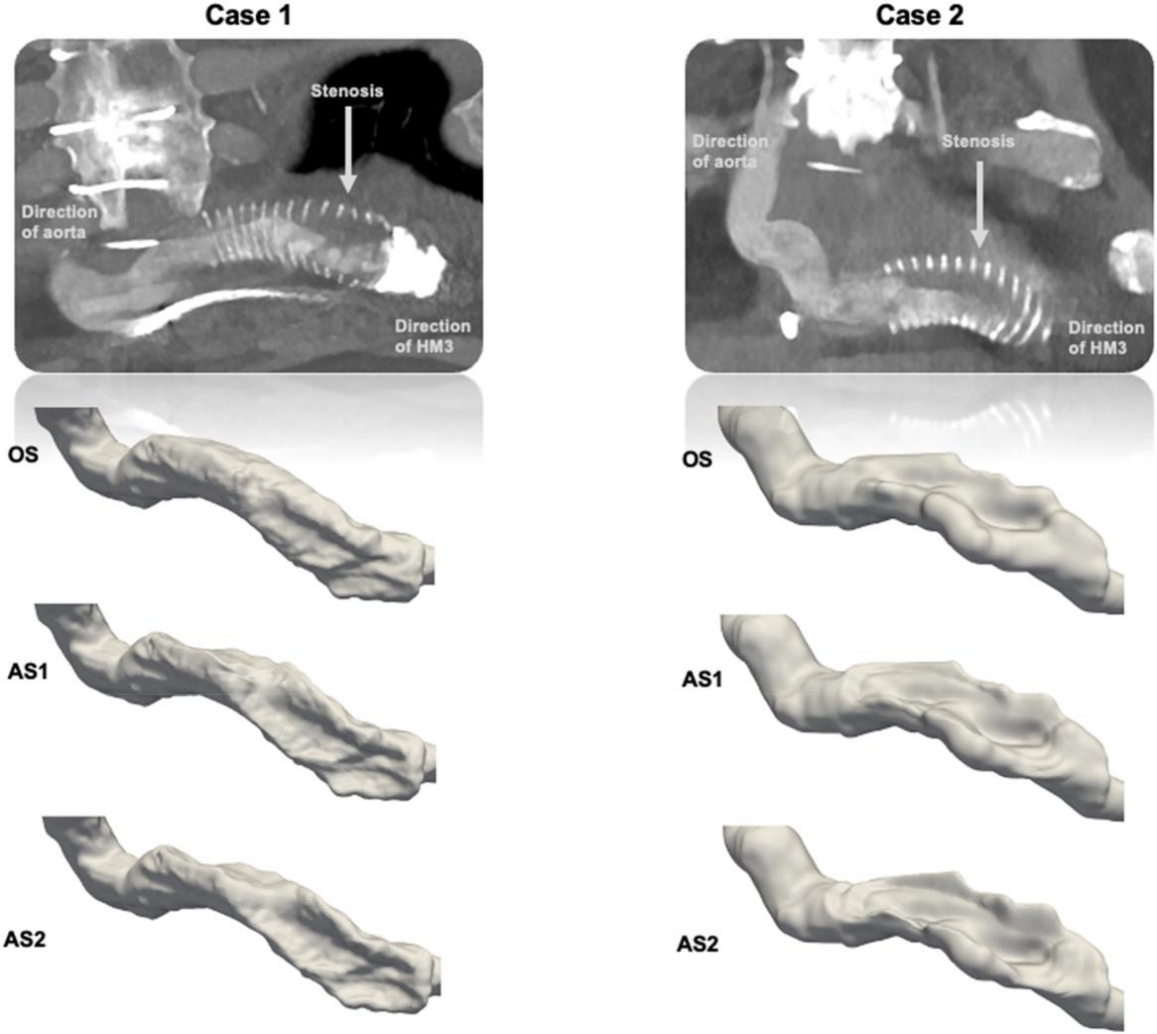
CT images displaying contrast-filled stenosed outflow graft lumen and the surrounding bend relief in the two cases (above). The three resulting outflow graft surfaces are demonstrated. OS, original stenosis; AS1, augmented stenosis 1; AS2, augmented stenosis 2.

The graft geometries from the two cases were segmented semi-automatically using ITK-SNAP 4.0 (Utah, PA, USA). To further investigate the effect of stenosis severity, the extracted geometries were virtually stepwise linearly deformed in Autodesk Meshmixer 3.5 (San Rafael, CA, USA) into two additional geometries for each case so that three stenosis severities per case were studied: original stenosis without any deformation (OS), augmented stenosis 1 (AS1), and augmented stenosis 2 (AS2) (see *Figure 1* and *Table 1*).

**Table 1:** Narrowest CSA and percentage reduction

Stenosis	Case 1 (mm^2^)	Case 1 (%)	Case 2 (mm^2^)	Case 2 (%)
OS	173	28	165	31
AS1	136	43	130	46
AS2	116	52	93	61

The narrowest CSA as absolute values (mm^2^) and per cent luminal reduction (%) for the different stenosis severities.

OS, original stenosis; AS1, augmented stenosis 1; AS2, augmented stenosis 2.

### Numerical model

The numerical haemodynamic evaluations were performed with computational fluid dynamics (CFD) using ANSYS CFX 2022 R2 (Canonsburg, PA, USA). Inlet boundary conditions were three different inlet flow rates (3.5, 4.5, and 5.5 L/min), which are in the normal flow range through the HM3. A constant static pressure boundary condition of 100 mmHg was set as reference pressure in the aorta. Large eddy simulation (LES), which is a high-fidelity scale-resolving turbulence model, was used to resolve large parts of the turbulent features in the flow. This approach to simulation of stenotic flows with LES has yielded results that agree well with other experimental measurement methods such as laser Doppler velocimetry (LDV)^17^ and MRI.^13^ The mesh size was on the order of 5.9–8.2 million cells, depending on geometry, with finer resolution near the wall and in regions where turbulent and disturbed flow were expected. Mesh independency tests were performed with mesh sizes from 2.5 to 12.5 million cells, and a difference of <0.1 mmHg was observed in the time-averaged pressure levels between the two finest mesh sizes. The flow was simulated as time resolved with a constant inflow, and velocity, pressure, and turbulent statistics were sampled over 6 s of flow time to yield statistically converged average values. The numerical schemes were second-order accurate and time step size was 5e^−5^ s. The density was set to 1060 kg/m^3^ and the viscosity to a constant value of 3.5e^−3^ Pa·s, i.e. a Newtonian fluid, as non-Newtonian effects could be ignored due to high shear levels in the fluid. The outflow graft and stenotic region were assumed to be rigid and fixed in space. The artificial pulse in the HM3 that periodically changes the LVAD rotor speed was not included in the simulation. For each patient, three different stenosis severities were assessed, each at three different inlet flow rates, yielding 18 different flow simulations in total.

### Haemodynamic parameters

Although pressure drop is directly associated with tissue perfusion and objective symptoms, blood flow velocity and turbulence development are crucial parameters for understanding pressure. Accordingly, the pressure profile across the stenosis was assessed by quantifying the average pressure in 10 evenly distributed (5 mm apart) cross-sectional planes over the stenotic graft segment. The pressure gradient was defined as the difference between maximum mean pressure and the reference pressure in the aorta. The pressure gradients were compared among the different stenotic severities and device flow rates. Furthermore, blood velocity profiles over the stenoses were qualitatively assessed by quantifying the velocity magnitude in evenly distributed cross-sectional planes over the stenotic graft region. The maximum velocity in the graft was compared between levels of stenosis and device flow rates. Furthermore, by using Reynolds decomposition to decompose the velocity signal into a mean and a fluctuating component, the amount of turbulent fluctuations can be described by the root mean square of the difference of instantaneous velocity and mean velocity:


$$u^{\prime }=\;\sqrt{\frac{1}{N}\sum_{i=1}^{N}(u_{i}-\;{\bar{u)}}^{2}}$$

where *N* is the number of samples in the signal. It is then possible to define the turbulent kinetic energy (TKE) as:


$$\mathrm{TKE}=\;\frac{1}{2}\rho (u^{\prime 2}+\upsilon^{\prime 2}+w^{\prime 2})$$

where $u^{\prime }$, $\upsilon^{\prime }$, and $w^{\prime }$ are the fluctuating velocity components and *ρ* the density.^18^ TKE is a measure of the turbulent fluctuations in the flow and was quantified, visualized, and compared between stenotic severities and device flow rates.

### Statistical evaluation

The level of significance was set at *P* < 0.05. Correlations between extracted parameters and stenosis severities and device flows rates were examined using Pearson’s correlation test. Both the correlation coefficient (*R*) and the calculated *P*-value are reported in the results.

### Ethical approval

The two cases were identified within an ongoing clinical trial (NCT 06115096). The overall research project has been approved by the Swedish ethics review authority (reference number: 2022-06934-01). All research subjects have provided written consent.

## Results

### Radiological evaluation

**Figure 2:**
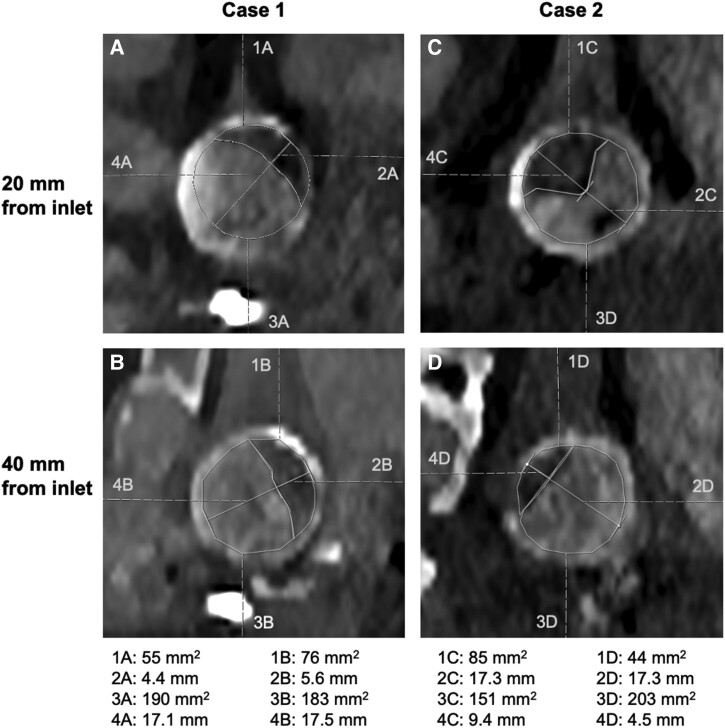
Radiological measurements of CSA and lumen diameter at 20 and 40 mm distal to the graft inlet. Values of areas and diameters are displayed beneath. (*A*) Case 1, 20 mm from the graft inlet. (*B*) Case 1, 40 mm from the graft inlet. (*C*) Case 2, 20 mm from the graft inlet. (*D*) Case 2, 40 mm from the graft inlet.

Visual inspection of the stenotic outflow graft by two experienced radiologists in consensus identified stenosis measurement sites at 20 and 40 mm from the graft inlet in both cases. At 20 mm distal to the graft inlet, Case 1 displayed a 24% lumen diameter reduction (4 mm/17 mm) and 22% CSA reduction (54 mm^2^/244 mm^2^). Case 2 had a 53% lumen diameter reduction (9 mm/17 mm) and 36% CSA reduction (85 mm^2^/236 mm^2^). In measurements performed at 40 mm distal to the graft inlet, Case 1 displayed a 35% lumen diameter reduction (6 mm/17 mm) and 29% CSA reduction (76 mm^2^/259 mm^2^). Case 2 showed 35% lumen diameter reduction (6 mm/17 mm) and 18% CSA reduction (44 mm^2^/246 mm^2^). The choice of the measurement sites was made by the radiologists and necessarily may not have encountered the most stenotic sites along the outflow graft. Radiological measurements are detailed in *Figure 2*.

### Pressure

**Figure 3:**
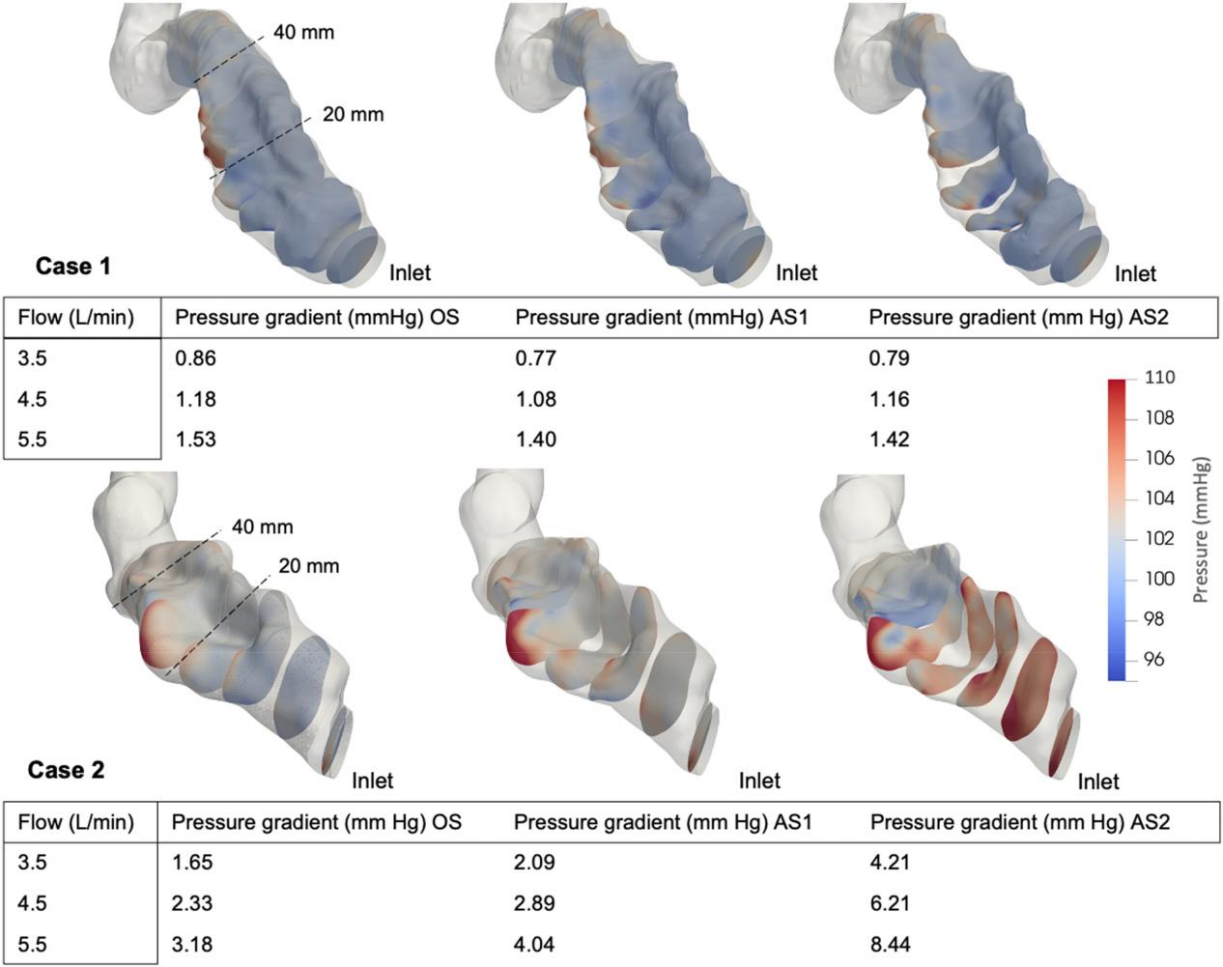
Pressure (mmHg) visualized in 10 cross-sectional planes over the stenotic segments at 5.5 L/min. Values of pressure gradients for each stenosis severity and device flow are displayed beneath. OS, original stenosis; AS1, augmented stenosis 1; AS2, augmented stenosis 2.

**Figure 4:**
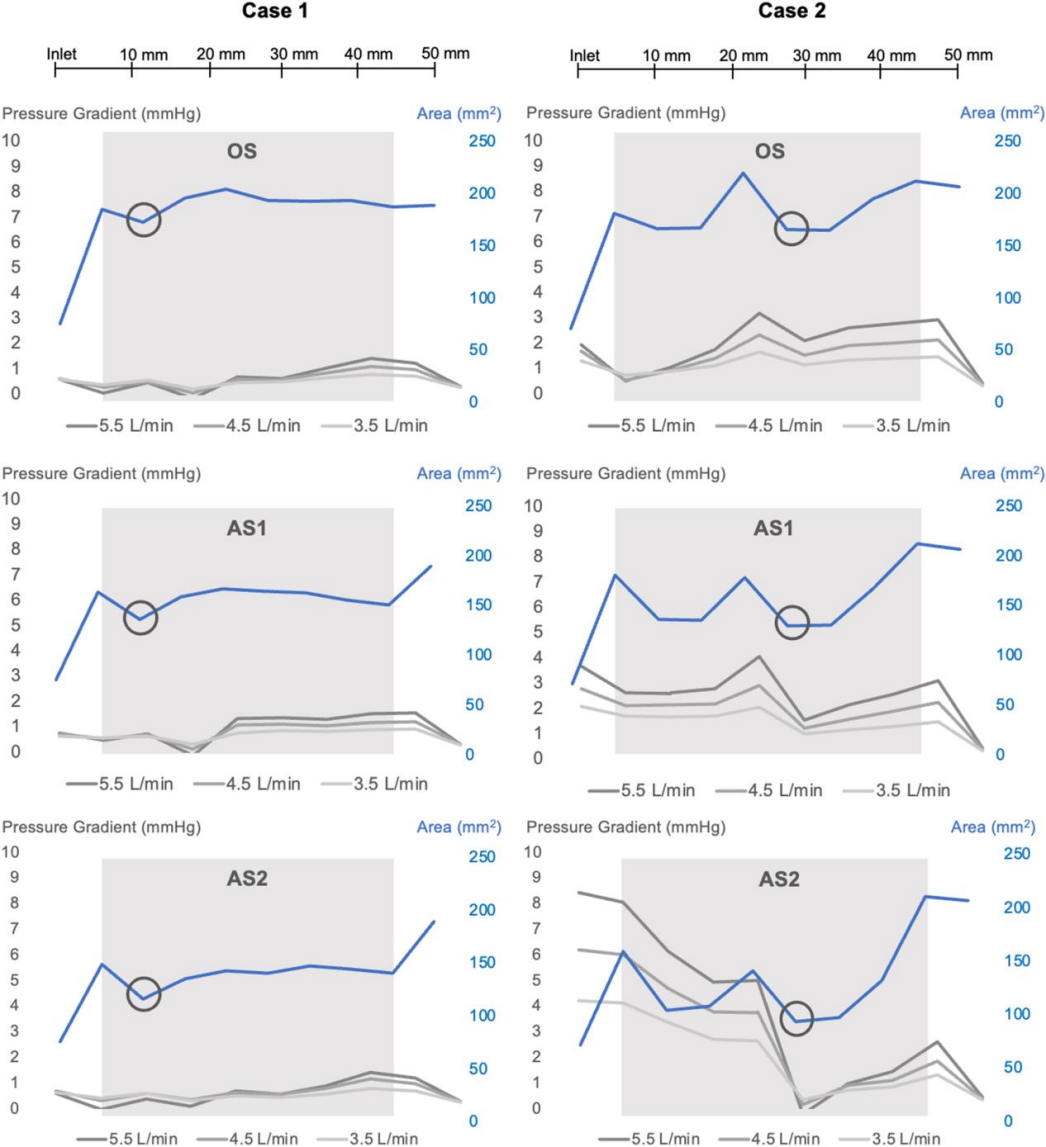
Average pressure gradient (mmHg) along the outflow graft for the different stenosis severities, depending on device flow (L/min) and in relation to CSA (mm^2^). OS, original stenosis; AS1, augmented stenosis 1; AS2, augmented stenosis 2. Areas of light grey indicate the length of the stenosis, and black circles indicate the narrowest point within the stenotic segment.

By qualitative assessment, the highest pressures occurred along the outer anterior curvature of the stenosed outflow graft, seen most prominently in Case 2. For Case 1, minimal changes in pressure gradient (<2 mmHg) were noted despite increasing stenosis and flow. In Case 2, the pressure profile was more affected, with a pressure gradient of 8.44 mmHg with the greatest stenosis (AS2) and highest flow setting. Case 2 showed a significant correlation between pressure gradient and stenotic severity (*P* = 0.01; *R* = 0.77), but Case 1 did not (*P* = 0.79; *R* = 0.10). There was a significant correlation between device flow rate and pressure gradient in Case 1 (*P* < 0.001; *R* = 0.98) but not in Case 2 (*P* = 0.16; *R* = 0.51). The mean pressure profiles corresponding to stenosis severities, CSA, and device flow are displayed in *Figures 3* and *4*.

### Blood flow velocity

**Figure 5:**
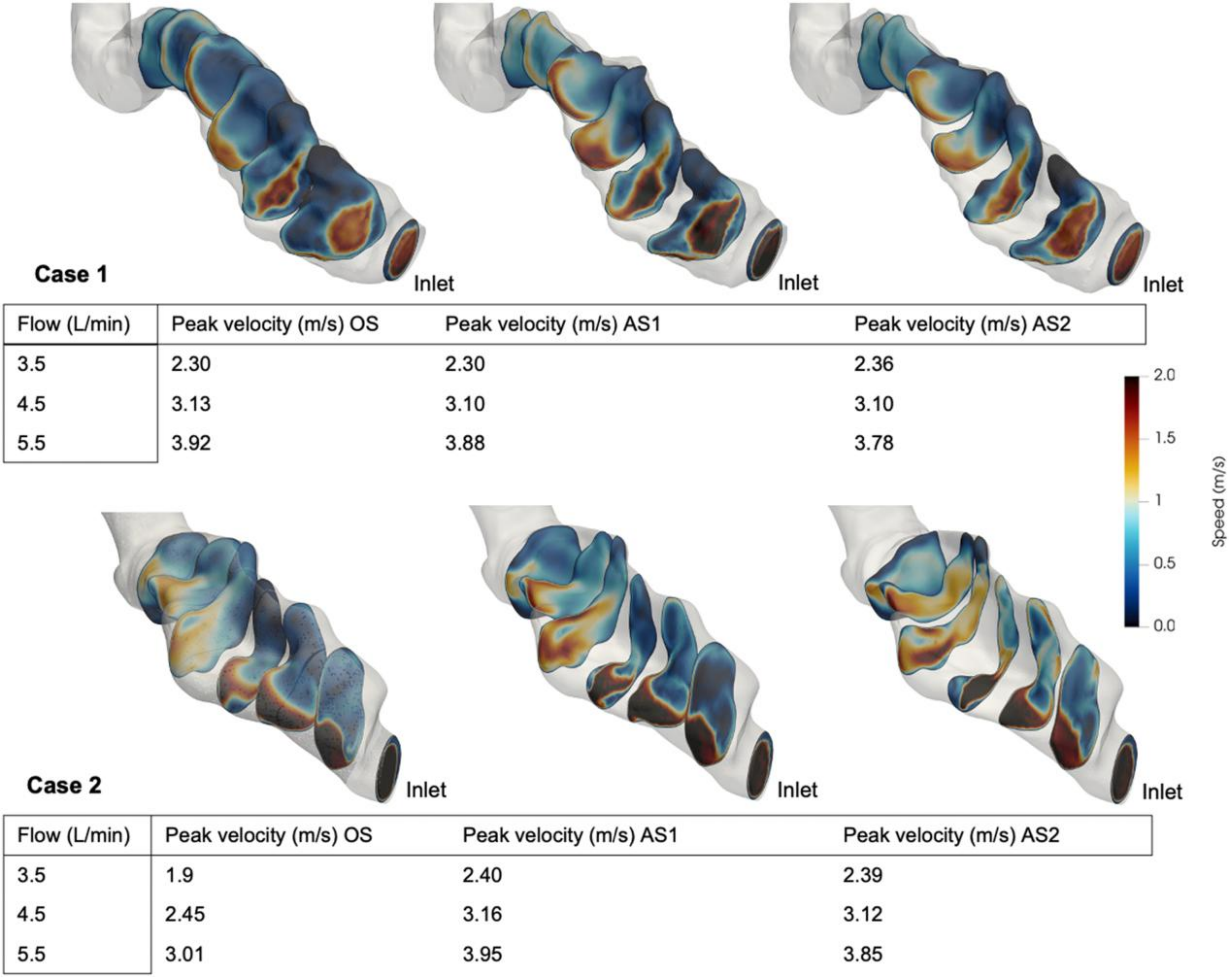
The mean magnitude of velocity in seven cross-sectional planes over the stenotic segment at 5.5 L/min is demonstrated in the images. Values of peak velocity (m/s) corresponding to stenosis severity and device flow (L/min) are shown. OS, original stenosis; AS1, augmented stenosis 1; AS2, augmented stenosis 2.

By qualitative assessment, the intra-graft blood flow was oriented to the outer anterior curvature of the outflow graft and the highest velocities were located there. This is clearly seen in Case 2 (*Figure 4*). There was a strong correlation between device flow and maximum blood flow velocity in both cases (Case 1: *P* < 0.001; *R* = 1.0; Case 2: *P* = 0.003; *R* = 0.86) but no significant correlation between stenosis severity and maximum velocity (Case 1: *P* = 0.95; *R* = 0.02, Case 2: *P* = 0.27; *R* = 0.41). The maximum velocity corresponding to the different severities of stenosis and input flows and the mean magnitude of velocity in seven CSA planes over the stenoses at 5.5 L/min are displayed in *Figure 5*.

### TKE

**Figure 6:**
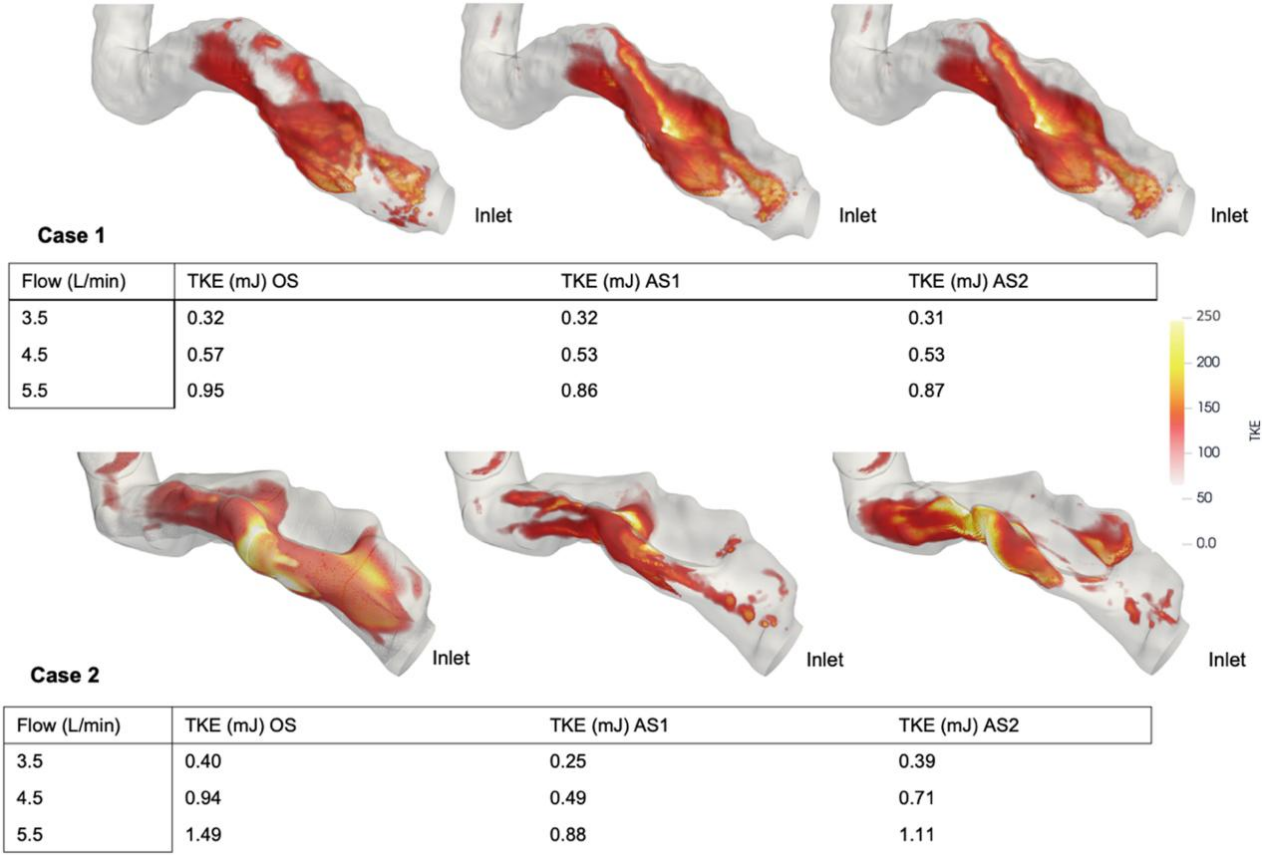
TKE (mJ) for the different stenosis severities and device flow (L/min) are shown. Images are acquired at 5.5 L/min device flow. Values < 75 are transparent to better highlight the higher values. OS, original stenosis; AS1, augmented stenosis 1; AS2, augmented stenosis 2.

By qualitative assessment, the highest TKE levels were located in the outer anterior curvature of the outflow graft in the middle of the stenotic region. Both cases showed a significant correlation between TKE and increased device flow rate (Case 1: *P* < 0.001; *R* = 0.99; Case 2: *P* = 0.002; *R* = 0.87). There was no significant correlation between TKE and stenotic severity (Case 1: *P* = 0.84; *R* = 0.077; Case 2: *P* = 0.57; *R* = 0.22). The magnitude of TKE for each stenotic severity and flow rate are displayed in *Figure 6*.

### Correlations with pressure gradients

**Figure 7:**
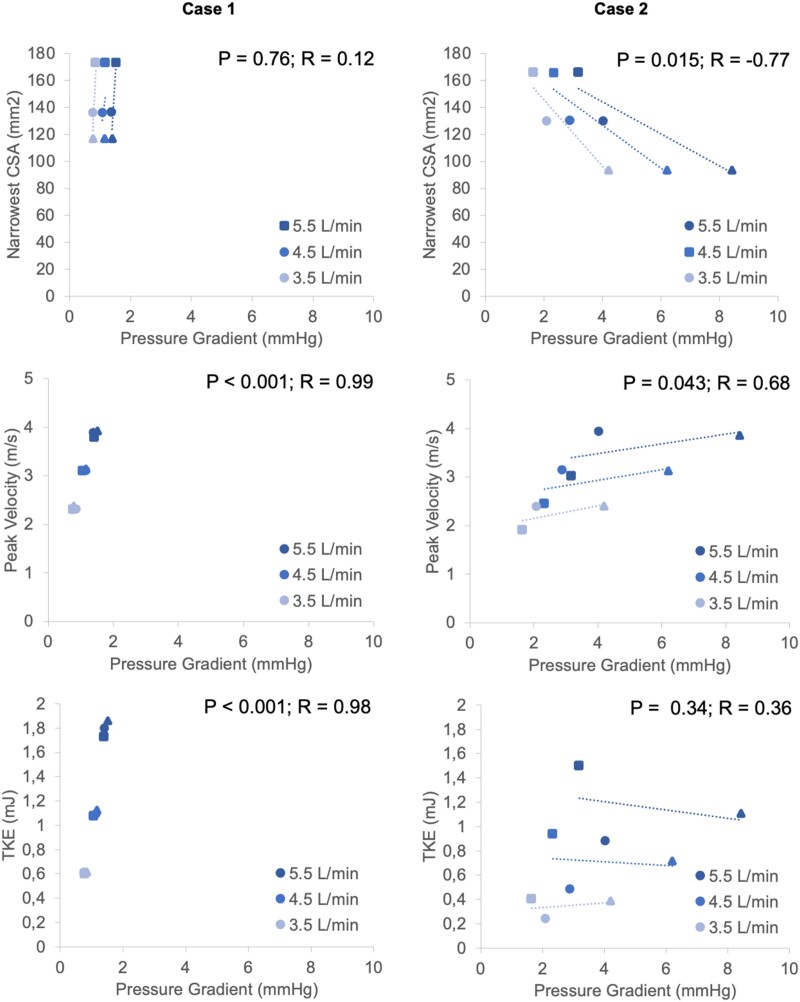
Correlations between pressure gradient (mmHg) and narrowest CSA (mm^2^), peak blood flow velocity (m/s), and TKE (mJ) among both different flow rates and stenosis severities. OS, original stenosis, squares; AS1, augmented stenosis 1, circles; AS2, augmented stenosis 2, triangles.

Case 2 showed a significant correlation between pressure gradient and narrowest CSA (*P* = 0.0015; *R* = −0.77), but Case 1 did not (*P* = 0.76; *R* = 0.12). Both cases showed a significant correlation between pressure gradient and peak blood flow velocity (Case 1: *P* < 0.001; *R* = 0.99; Case 2: *P* = 0.043; *R* = 0.68). Case 1 showcased a significant correlation between pressure gradient and TKE (*P* < 0.001; *R* = 0.98) but Case 2 did not (*P* = 0.34; *R* = 0.36). Correlations between pressure gradient and narrowest CSA, peak blood flow velocity, and TKE are visualized in *Figure 7*.

## Discussion

PCCT provides very high-resolution images, even in the presence of metal HM3 components. Utilizing these images, we performed a novel high-fidelity numerical assessment on two patients with radiologically identified HM3 outflow graft stenoses. We compared patient-specific anatomy with virtually exaggerated stenoses under conditions of three device flow rates. Despite clinical concern for significant stenosis and the recent Class 1 device recall, neither the original geometries nor the virtually augmented stenoses were associated with high-pressure gradient or TKE. Notably, there was poor correlation between radiological assessment and pressure gradient; while the radiologically assessed area reduction was similar in both patients, the haemodynamic impact with respect to pressure gradient and turbulence across the tested flow rates differed.

The subtle pressure gradients measured in this study of two clinically stable, asymptomatic patients contrast with previously described clinical cases where stenoses were associated with severe and rapidly progressive clinical courses.^6,7^ In a clinical series of 20 patients (of which 3 with HM3) with clinically significant outflow graft obstruction, the mean gradient was 78 mmHg assessed with catheterization, declining to 10 mmHg after successful stenting.^19^ However, the study included both intrinsic and extrinsic aetiology of stenosis. The low gradients in this study’s subjects including the augmented stenotic geometries may be a consequence of insufficiently severe stenoses. A multi-centre study of 62 symptomatic and asymptomatic patients with eOGO^8^ defined significant stenosis as >25% area reduction, and a majority underwent interventions to relieve obstruction. The findings in our cases, with 29 and 36% area reductions but without clinical symptoms, suggest that the narrowest CSA alone cannot account for the pressure drop and haemodynamic impact. It is likely that both the extent of stenosis in the longitudinal direction (*Figure 3*) and the U-shaped lateralization of blood flow observed in this study collectively influence the ultimate haemodynamic impact of the eOGO. In other stenoses in the cardiovascular system, e.g. aortic stenosis, the irreversible pressure loss caused by energy dissipation in post-stenotic flow are well correlated with TKE.^20,21^ However, in this study, the measured TKE values are significantly lower than in studies of other significant stenoses,^13,20,21^ in line with the observed low-pressure gradients.

The mechanisms underlying the onset and progression of outlet graft stenosis are important to prevention of complications. In our haemodynamic simulations, it is evident that velocity, turbulence, and local pressure are lateralized to the outer anterior curvature of the stenosed graft. Might such flow lateralization facilitate the development of extrinsic compression on the opposite side of the graft where the local pressure against the graft wall is relatively lower? Microleakage into the space between the graft and the bend relief collar could, over time, accumulate preferentially around that lower pressure graft wall, eventually organizing into fibrinous material and distorting the inner posterior curvature of the graft. An outflow tract that generates a more central jet towards the proximal part of the outflow graft and/or a permeable bend relief might reduce the risk of this extraluminal compression.

Classification of the functional significance of extrinsic outflow graft stenoses is limited by the lack of non-invasive haemodynamic modalities. Haemodynamic numerical models such as applied here may play a significant role in investigations where cohorts are limited, flow conditions cannot be manipulated, or necessary invasive measurements are unavailable. By virtually deforming geometries or changing boundary conditions such as flow and heart rate, numerical models can aid in predicting the significance of stenoses over a range of conditions as well as the potential effect of interventions.

Numerical flow simulations inside a HM3 device have been compared with measurements in an *ex vivo* setting.^22,23^ Unfortunately, there is a lack of research and validation on *in vivo* data. Numerical simulations have been used to assess the outflow angle of the outflow graft into the ascending aorta.^24,25^ However, no validations for CFD have been shown on intra-graft haemodynamics. The accuracy of haemodynamic numerical models depends on model assumptions including boundary conditions and detailed geometry. Patient-specific boundary conditions may be difficult and sometimes impossible to obtain. In this study, the inlet velocity profile was assumed to be flat, while the centrifugal pump in the HM3 may result in a non-uniform velocity profile at the pump–graft interface. In addition, stenosis delineation was hampered by metal artefacts from the HM3 despite multiplanar reconstructions. This may have influenced the accuracy of segmentation and surface extraction as well as the radiological assessment. The artificial pulse in the HM3 was not mimicked in the simulations. It has been shown in previous studies that the artificial pulse improves washout from the device, which may explain the low incidence rates of pump thrombosis.^26^ However, the impact of the artificial pulse on haemodynamics during eOGO remains unknown. Lastly, there is no gold standard for assessment of haemodynamic severity of outlet graft stenosis, making cut-off values for haemodynamic significance challenging to determine.

On the basis of radiological images alone, outflow graft stenoses can raise strong clinical suspicion of significant risks for the patients. This study suggests that the geometries of the stenoses are too complex to be graded by maximal area reduction alone. The mortality following interventions for eOGO has been observed to be as high as 17%.^8^ Therefore, in the absence of patient symptoms or instability, vigilant clinical monitoring while remaining mindful of the potential for stenosis progression over time may be appropriate.

In conclusion, HM3 outflow graft stenosis identified on radiological surveillance in an asymptomatic patient may evoke clinical apprehension and prompt intervention. In this numerical study, virtual models of graft stenoses analysed with input from state-of-the-art PCCT suggest that the haemodynamic impact of the stenoses was low despite meeting used criteria for significant obstruction. The haemodynamic impact, defined by the pressure gradient over the stenosis and change with flow rate, was difficult to predict and easily overestimated using visual assessment and quantification of CSA reduction. Further refinement of radiological metrics and severity grading thresholds may be achieved with numerical modelling.

## Data Availability

The data underlying this article can be shared upon reasonable request from the corresponding author.
